# Author Correction: Boundary integral equation method for resonances in gradient index cavities designed by conformal transformation optics

**DOI:** 10.1038/s41598-021-81203-w

**Published:** 2021-01-08

**Authors:** Jung-Wan Ryu, Jinhang Cho, Soo-Young Lee, Yushin Kim, Sang-Jun Park, Sunghwan Rim, Muhan Choi, Inbo Kim

**Affiliations:** 1 Center for Theoretical Physics of Complex Systems, Institute for Basic Science (IBS), Daejeon, 34126 Republic of Korea; 2 Digital Technology Research Center, Kyungpook National University, Daegu, 41566 Republic of Korea; 3 School of Electronics Engineering, Kyungpook National University, Daegu, 41566 Republic of Korea; 4 KU-KIST Graduate School of Converging Science and Technology, Korea University, Seoul, 02841 Republic of Korea

Correction to: *Scientific Reports* 10.1038/s41598-019-55572-2, published online 23 December 2019

The Author Contributions section in this Article is incorrect.

“The late S.-Y.L. conceived the original idea. J.-W.R., S.-Y.L., M.C. and I.K. formulated the method. J.-W.R., J.C., Y.K. and S.-J.P. performed numerical calculation. J.-W.R., J.C., S.R., M.C. and I.K. wrote the manuscript. All authors analyzed the data, discussed the results and provided feedback for the manuscript.”

should read:

“The late S.-Y.L. conceived the original idea for this BEM. J.-W.R., S.-Y.L., M.C. and I.K. formulated the method. J.-W.R., J.C., Y.K. and S.-J.P. performed numerical calculation. J.-W.R., J.C., S.R., M.C. and I.K. wrote the manuscript. All authors analyzed the data, discussed the results and provided feedback for the manuscript.”

Also, this Article contains an error in the Conclusion section.

“In order to obtain the resonant mode functions in TCs designed by conformal TO, we have developed a pure BEM based on the fact that the Green’s functions of interior and exterior regions are known in the RV and the physical spaces, respectively, which are connected by a conformal mapping. For the verification of our BEM, we have respectively, which are connected by a conformal mapping. For the verification of our BEM, we have calculated resonant modes in limaçon-shaped TCs and compared those with the corresponding results from FEM.”

should read:

“In order to obtain the resonant mode functions in TCs designed by conformal TO, we have developed a pure BEM based on the fact that the Green’s functions of interior and exterior regions are known in the RV space and the physical space, respectively, which are connected by a conformal mapping. For the verification of our BEM, we have calculated resonant modes in limaçon-shaped TCs and compared those with the corresponding results from FEM.”

